# Motors vs. operators in simulated root canal shaping

**DOI:** 10.3389/fdmed.2025.1617425

**Published:** 2025-07-25

**Authors:** Kevin Hofpeter, Matthias Zehnder, Shengjile Deari

**Affiliations:** Clinic of Conservative and Preventive Dentistry, Division of Endodontology, Center for Dental Medicine, University of Zurich, Zurich, Switzerland

**Keywords:** endodontic motor, root canal, endodontic training block, Reciproc, instrumentation

## Abstract

**Introduction:**

The impact of contemporary endodontic motors on shaping outcomes has not been weighed against that of the motor operators.

**Materials and methods:**

One motor (X-Smart Pro+) specifically developed for the reciprocating files that were used (Reciproc Blue R25) was compared to three cordless counterparts, two of which lacked a designated reciprocation mode. Standardized J-shaped canals in bovine incisor roots were instrumented by four different operators, who were residents with similar levels of education and clinical experience. One reciprocating file per simulated root canal was used. The root canal models were pre-warmed and kept in a vice at 37°C in a water bath. The operators were instructed to instrument two simulated canals per motor in a random sequence, applying three pecking motions and alternating with 3% NaOCl irrigation. Instrumentation time was measured. Pre- and postoperative images obtained using a digital microscope were superimposed to assess canal transportation. Parametric tests (two-way ANOVA) were applied to weigh the overall effects of the motor and operator on instrumentation time and canal transportation. The impact of the motor and operator on the number of unwound flutes was explored using likelihood ratio tests. The level of significance was set at 5% (*P* < 0.05).

**Results:**

Operators had a highly significant (*P* < 0.001) impact on instrumentation time and file unwinding, while motors did not (*P* > 0.05). File unwinding was negatively correlated with instrumentation time (*P* < 0.001). There was no effect of either the motor or the operator on canal transportation (*P* > 0.05).

**Conclusion:**

Technological advancements in endodontic motors do not necessarily compensate for operator variability.

## 1 Introduction

In endodontics, there has been a continuous trend away from hand toward engine-driven root canal instrumentation (1). However, while there is a plethora of studies on files and file systems (2), investigations on endodontic motors have been few (3, 4). Motors specifically designed to run endodontic files have evolved significantly in recent years. Indeed, the option of one specific motor (ATR Vision, ATR, Pistoia, Italy), which allowed to program the reciprocating movement of endodontic files at defined angles, has enabled a whole new motion concept, in which the rotary files engage and disengage with the root canal wall at defined angles (5). Reciprocation has now become an industry standard. A survey in Switzerland showed that more than half of the recent dental school graduates used motorized reciprocating systems in general dental practice, even though these systems had not been taught at their schools at the time (6).

It would appear logical that a reciprocating movement, which involves a defined clockwise and a counterclockwise movement, requires more from a motor than simple unidirectional rotation. Moreover, the specific file movements are proprietary, and although data on file movements have been published (7), it remains unclear whether motors not specifically designed for a reciprocating file system can perform as well as a motor designed by the company manufacturing the instruments that it drives. The makers of the original reciprocating file systems (Dentsply Sirona, Charlotte, NC, USA) recently launched a motor that claims to be 21% and 14% faster at instrumenting resin training blocks than competitors when using one of their reciprocating or rotary file systems, respectively. This is stated to be due to a patented sensorless system for 360° speed and torque feedback. While these claims potentially hold true in a standardized environment (4), there is another element that has not received the attention it deserves in endodontic instrumentation studies: the impact of the operator. Studies in 3D-printed teeth have shown that between operators, there can be a considerable difference in shaping outcomes, including the time required to instrument a simulated root canal (8). Most importantly, clinical studies have shown a difference in endodontic treatment quality and outcome based on the operators' gender and educational background (9). However, motors can potentially modulate operator shortcomings. Depending on operator skills, there seems to be an impact of motor settings on file separation (10). However, the influence of motors vs. operators has not been tested with reciprocating files in a contemporary setting.

The goal of this study was to compare four currently marketed and popular endodontic motors driving a reciprocating file in a controlled setting, which was a standardized J-shaped simulated root canal in bovine dentin (11). The primary outcome that was assessed was preparation time, i.e., time to reach working length. The null hypothesis tested was that there was no difference in instrumentation time between the motors under investigation. The secondary outcomes were the centering ratios of the preparations and file unwinding as a sign of torsional overload (12). Four residents in Conservative Dentistry were the operators. The impact of the motor on the outcomes under investigation was weighed against that of the operator.

## 2 Materials and methods

### 2.1 Models

The models that were used have been described in detail elsewhere (11). They correlated to the classic size 15 J-shaped resin training block (Dentsply Maillefer, Ballaigues, Switzerland). Canals were milled into flattened and polished root halves (Planopol-2, Struers, Copenhagen, Denmark) in bovine incisor roots embedded in methyl methacrylate (Paladur, Kulzer, Hanau, Germany) using a precision milling setup (GS600/5-FDT, Alzmetall, Altenmarkt, Germany). The two parts were reassembled in a custom-made vice and kept in a water bath at 37°C for instrumentation (see below).

### 2.2 Motors and operators

The motors and settings that were used are summarized in Table 1. All these motors came to market in 2023. One, the X-Smart Pro+ (Dentsply Sirona), was specifically designed to drive the reciprocating files under investigation. This motor features an external battery and a touch screen connected to the handpiece. It has proprietary settings for the reciprocating file that was used (Table 1). The other three motors under investigation were cordless. Two [the Tri Auto ZX2+ (Morita, Tokyo, Japan) and the EndoPro Ai1 (Brasseler, Savannah, GA, USA)] did not feature an explicit reciprocating mode. Instead, they had a rotating mode, in which reciprocation was triggered above a defined torque value. Therefore, the torque control values were set to the lowest level possible to trigger reciprocation, and the reciprocating movement was entered as described in Table 1. This is commonly done by dentists who do not have a cordless motor with a designated reciprocation mode. The fourth motor (Elements Connect, Kerr, Brea, CA, USA) did feature a specific reciprocation mode, yet the nominal speed and torque settings were proprietary.

**Table 1:** Motors used in this study and their settings.

Motor	Torque setting (N·cm)	Angle ccw/cw (°)	Nominal speed (rpm)
X-Smart Pro+ (Dentsply)	4	Proprietary	Proprietary
Tri Auto ZX2+ (Morita)	0.2^a^	150:30^b^	300
EndoPro Ai1 (Brasseler)	0.4^a^	150:30	300
Elements Connect (Kerr)	Proprietary	150:30	Proprietary

ccw, counterclockwise; cw, clockwise.

^a^ Used to trigger reciprocation.

^b^ From Ref.

The operators were four residents in Conservative Dentistry, three women and one man, aged 26–31 years. They were familiar with the reciprocating system that was used in this study (Reciproc Blue R25, Dentsply VDW, Munich, Germany) and used it clinically, but not in conjunction with any of the motors under investigation.

### 2.3 Power analysis

The primary outcome was preparation time in seconds according to the motor that was used. Pilot studies in resin training blocks with a single operator resulted in an effect size of 0.9. With four groups, an alpha-type error of 5%, and 0.95 power, a total sample size of 28 was calculated (*n* = 7, G*Power 3.1, Heinrich Heine University, Düsseldorf, Germany). Therefore, each operator was asked to prepare two simulated root canals each, resulting in a total sample size of 32 (*n* = 8).

### 2.4 Canal preparation

The operators were asked to prepare the simulated root canals. They were informed that the time to do so would be measured and that shaping outcomes would be considered in the study. The sequence in which the motors were used by each operator was randomized. To mask the motor brands, the motors/handpieces were covered with opaque adhesive tape. The operators were asked to perform three pecking motions and then irrigate the simulated canal with 1 mL of a 3% NaOCl solution (Hedinger, Stuttgart, Germany) using a polypropylene tip (IrriFlex, Produits Dentaires, Vevey, Switzerland). Subsequently, files were cleaned in a clean stand (Dentsply Sirona) before the operator performed the next three pecking motions. The preparations were performed in fully hydrated dentin (the models had been kept in tap water prior to the experiment). The models were pre-warmed and kept in a water bath at 37°C to simulate clinical conditions (13).

### 2.5 Outcome assessments

The time to reach full working length (15 mm) was recorded using a stopwatch by one investigator (KH) sitting next to the operator. The effective instrumentation time, i.e., the amount of time the files were used in the simulated canal, was recorded. The time taken to irrigate the simulated canals and clean the files was not considered (14).

Canal transportation was assessed in the disassembled root half containing the milled canal using a digital microscope (VHX-2000, Keyence, Osaka, Japan). The centering ratios at 0.5, 1, and 2 mm from the working length were averaged and compared between the four motors (15). Because transportation occurred in both directions, i.e., to the inside and the outside of the curve, absolute values were used for statistical comparison (11).

**Figure 1:**
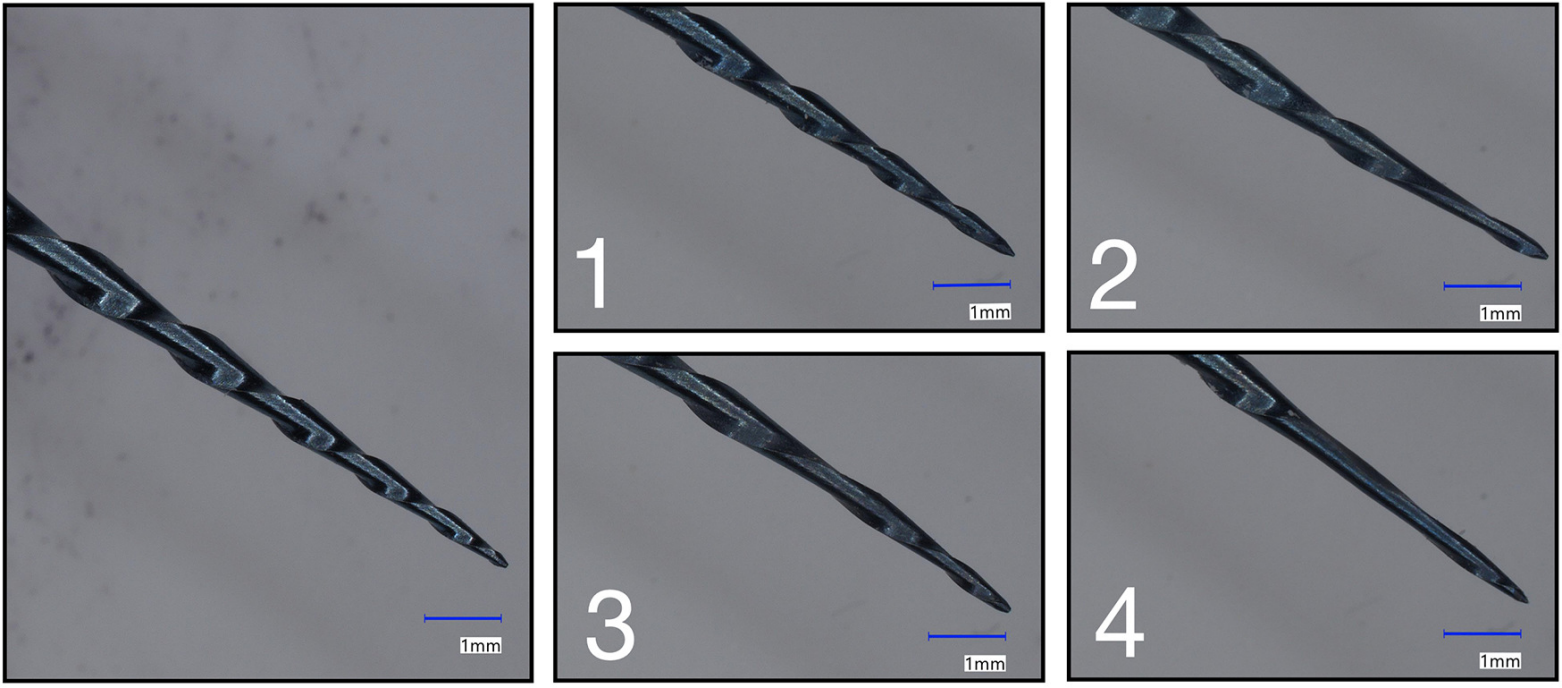
Digital microscopy images of the file tips before (left) and after use. In the right panels, representative examples of 1, 2, 3, and 4 unwound flutes are shown (according to the numbers in the panels).

Any unwinding of the files was also assessed using the digital microscope (VHX-2000) by superimposing an image of a virgin R25 file on that of a used file (16). To this end, files were embedded in a custom-made holder so that their flutes could be compared. The number of unwound flutes was reported (Figure 1).

Canal transportation and file unwinding were assessed by one of the authors (SD), who was blinded to the group allocation of the specimens under investigation.

### 2.6 Data presentation and analysis

Data that were distributed evenly (Shapiro–Wilk test) are presented as means and standard deviations. Parametric tests (two-way ANOVA) were applied to test for overall effects of motor and operator (and their interaction) on those outcomes. Differences within groups were explored using one-way ANOVA and Tukey's honestly significant difference (HSD) test. The impact of motor and operator on the number of unwound flutes (ordinal variable) was explored using likelihood ratio tests. The level of significance was set at 5% (*P* < 0.05).

## 3 Results

### 3.1 Instrumentation time

Reciprocating files driven by the X-Smart Pro+ motor required the least time to reach the working length, with 57 ± 17 s. This was roughly 20% quicker than the Tri Auto ZX2+ motor with 73 ± 20 s or the Elements Connect motor with 72 ± 31 s. However, the differences between the motors failed to reach statistical significance (Table 2). This was explained by the high variance, which was due to the differences between the operators. The influence of the operator (irrespective of the motor they used) was highly significant, and there was no significantly different effect of the motor depending on the operator (Table 2). Two operators were significantly (*P* < 0.05) faster at instrumenting the simulated canals compared to their two colleagues. Their instrumentation times were 46 ± 13 s and 55 ± 13 s vs. 80 ± 18 s and 80 ± 23 s, respectively.

**Table 2:** Effect tests (two-way ANOVA) of “motor” and “operator” on instrumentation time.

Source	DF	F ratio	*P*-value
Motor	3	2.252	0.1216 (NS)
Operator	3	10.199	0.0005
Motor × operator	9	1.300	0.3098 (NS)

DF, degrees of freedom; NS, not significant.

### 3.2 Canal transportation

The mean centering ratios were rather similar between the four motors under investigation. There was no significant impact of the motor or the operator on this outcome (Table 3).

**Table 3:** Two-way ANOVA of the effect of “motor” and “operator” on simulated canal transportation (centering ratio).

Source	DF	F ratio	*P*-value
Motor	3	0.3349	0.8003 (NS)
Operator	3	3.0187	0.0605 (NS)
Motor × operator	9	2.2855	0.9691 (NS)

DF, degrees of freedom; NS, not significant.

### 3.3 Unwinding of instruments

**Figure 2:**
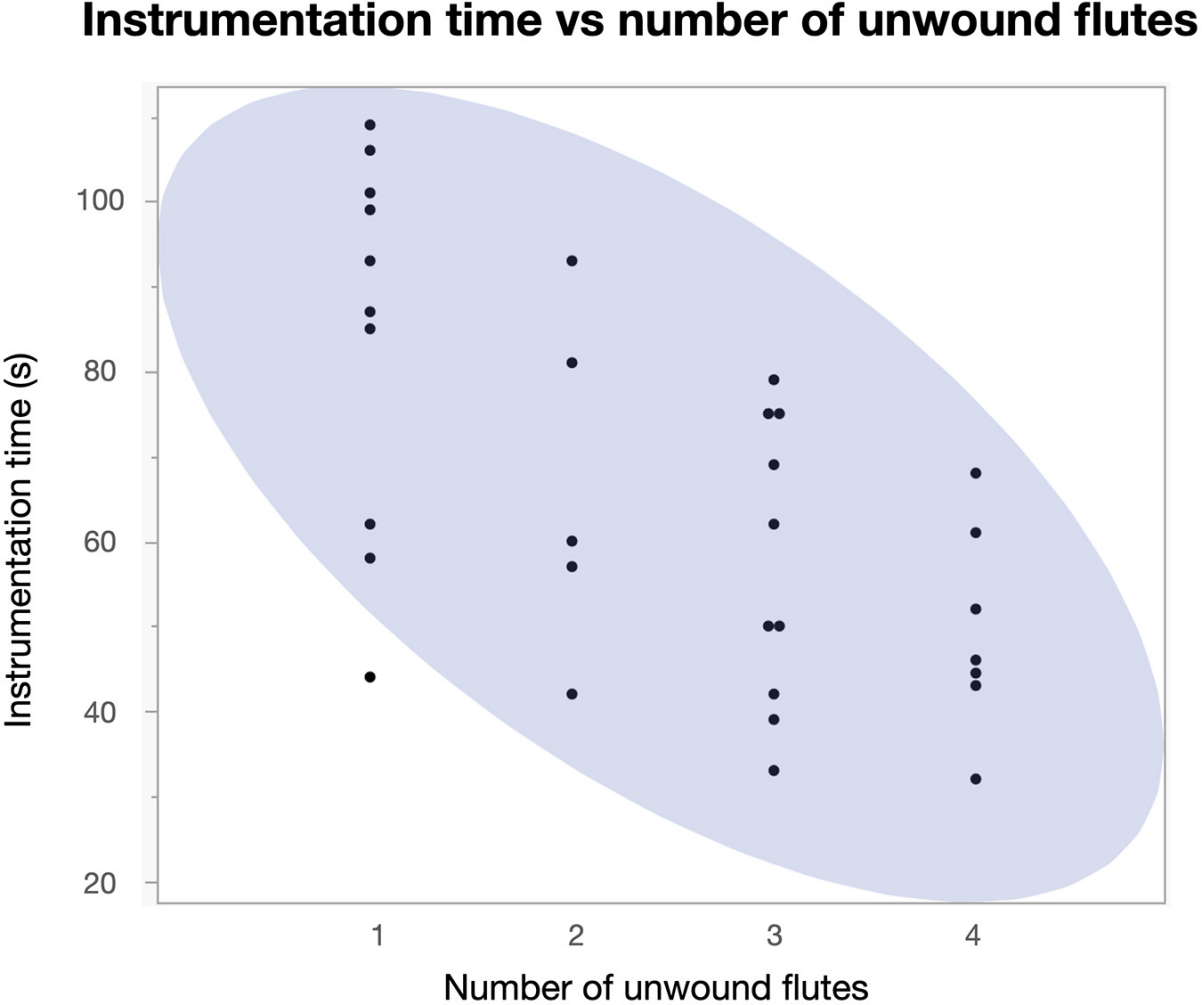
Dot plot including bivariate normal density ellipse (90%) depicting the correlation between instrumentation time and the number of unwound flutes after using the instruments.

No file fractured during the experiments. File unwinding was observed with all motors and operators, from 1 to 4 flutes. There was no difference according to motor, while the operator had a highly significant effect (Table 4). This effect could also be explained by the time taken for instrumentation: there was a strong negative correlation between instrumentation time and the number of unwound flutes (Figure 2, *P* < 0.05).

**Table 4:** Likelihood ratio tests of the effect of “motor” and “operator” on the number of unwound flutes in the reciprocating instruments after use.

Source	DF	LR chi-square	*P*-value
Motor	3	3.0355 × 10^−8^	1.0000 (NS)
Operator	3	59.0176	<0.0001
Motor × operator	9	13.0260	0.1614 (NS)

DF, degrees of freedom; LR, likelihood ratio; NS, not significant.

## 4 Discussion

The choice of endodontic motors has been discussed in endodontology with regard to the preparation time, centering ability of the driven instrument, and safety (file unwinding). However, despite technical developments, there is a lack of data on the influence of the operator in endodontic instrumentation. This study revealed some interesting new information regarding endodontic motors and their impact on shaping outcomes in a standardized root canal model in bovine teeth. The most interesting (and somewhat unexpected) finding was the difference between the operators, which obscured the other possible differences that potentially existed between the motors under investigation.

The current findings were obtained in a controlled experimental setting under *in vitro* conditions. This entails some limitations with regard to clinical applicability. Bovine teeth were used instead of human counterparts, with slight differences in micro-morphology, chemical composition, and physical properties (17). In addition, the experimental setting lacked clinical challenges such as workplace ergonomics (e.g., access to the oral cavity, view next to the motor handpiece) or variance in canal anatomy. This further limits the transferability of our results to clinical practice. For these reasons, the current data do not necessarily suggest that all the motors under investigation were similarly useful for the purpose of reciprocating root canal instrumentation. Moreover, there are other motor features that were not investigated or considered in this study, such as user-friendliness, automated length control, battery life, durability, speed, and real-time torque control, to name a few. Further studies are required to investigate these characteristics. Nevertheless, the current study revealed some interesting observations, which are discussed below.

The design of this study was closer to a clinical investigation with multiple operators than a classic endodontic bench-top study, in which one operator typically performs all the treatments. However, some investigations on the topic of endodontic motors and also on instruments compared individual operators with different levels of experience (10, 18). A study on reciprocating instrumentation in resin training blocks with 10 dental students with no experience in instrumentation vs. 10 dentists showed that the former took significantly longer to reach the shaping goal than the latter. However, the shaping outcomes were similar between the two groups (19). The operators in the current study were from the same clinic, in a similar age bracket, and shared comparable levels of clinical experience. It could thus be concluded that their approach to a well-defined simulated clinical scenario, such as the instrumentation of a curved canal, should result in a more uniform outcome. This was clearly not the case. In theory, a sophisticated motor should counteract operator shortcomings and variance. Features such as torque control have been shown to reduce cyclic fatigue in motor-driven endodontic instruments in the hands of one operator (3). However, the newly released motor under investigation (X-Smart Pro+, Dentsply Sirona), which was specifically designed to drive the proprietary file systems, including Reciproc Blue, did not speed up canal instrumentation significantly compared to the cordless motors under the conditions of this study. Nevertheless, there was a clear tendency toward reduced instrumentation time with the X-Smart Pro+ motor (Table 2). Moreover, and perhaps more importantly, file unwinding as a sign of torsional overload was not prevented, but remained a function of instrumentation time (Figure 2). This can only be interpreted in one way: the more pressure the operator exerted on the handpiece, the shorter the time to reach the working length, yet this led to more stress placed on the reciprocating instruments despite the obvious difference in the torque settings of the motors (Table 1).

The instrumentation time measured in this study was comparable to that of root canals in extracted human molars and premolars instrumented with reciprocating size-25 files, which ranged between 38 ± 14 s and 82 ± 25 s (20, 21).

Canal transportation in this study was lower than in a comparable earlier investigation (11). The averaged centering ratio (multiplied by 10) in the present study was 1.1 ± 0.9. This is considerably lower than that in a historic control, in which bovine dentin models of the same design were instrumented with the original, austenitic, Reciproc R25 files (11). The averaged ratio in that study was 2.3 ± 1.2 when water was used as an irrigant. It may therefore be suspected that transportation appears to be a function of metallurgical file properties and not only geometrical file design, even though there have been some conflicting findings regarding this issue with the reciprocating files under investigation (22). This could be a topic for future investigations.

File unwinding is a sign of torsional overload (1). De-Deus et al. only reported one deformed Reciproc Blue R25 instrument after instrumenting 100 extracted human mandibular molars (23). However, they did not specifically focus on file unwinding in their study. The simulated root canal used in this study represents a relatively hard-to-instrument curved and narrow canal, which may further explain the difference.

Technological advancements in endodontic motors aim to improve treatment outcomes and compensate for operator variability. As discussed above, there is a paucity of research in endodontic motors. Future studies should evaluate relevant motor characteristics not investigated here, such as ergonomics, real-time torque control, battery life, and user-friendliness. However, and importantly, the influence of practical skill and technique shows that even the most advanced motor may not be capable of replacing the need for adequate training and experience. Therefore, future studies should also analyze how operator characteristics can be influenced to improve clinical outcomes (9).

## 5 Conclusions

The current study failed to reveal a significant impact of the motor used for reciprocating instrumentation on the outcomes under investigation. This was potentially due to the high impact of the operators, who differed significantly in their performance. The current findings challenge the assumption that technological advancements in motors necessarily compensate for operator variability.

## Data Availability

The raw data supporting the conclusions of this article will be made available by the authors, without undue reservation.
